# Applying and validating the METUX model in Chinese higher education: a psychometric assessment of AI-based need satisfaction scales

**DOI:** 10.3389/fpsyg.2026.1822374

**Published:** 2026-07-03

**Authors:** Wei Zhang, Tianhua Yin, Sha Ouyang, Fengmei Zhu, Xuepeng Guo, Jing Xiong, Xiaolu Dong

**Affiliations:** 1 School of Marxism, Ningbo University of Finance and Economics, Ningbo, Zhejiang, China; 2 Faculty of Educational Studies, University Putra Malaysia, Sri Kembangan, Malaysia; 3 Shaanxi Railway Vocational and Technical College, Xi’an, Shaanxi, China; 4 Xi’an MingDe Institute of Technology, Xi’an, Shaanxi, China; 5 Xianyang Qidi Middle School, Xianyang, Shaanxi, China

**Keywords:** AI, Chinese, METUX model, need satisfaction scales, validation

## Abstract

As artificial intelligence (AI) technologies become increasingly embedded in higher education, understanding students’ psychological experiences with these tools is essential. Drawing on the METUX model (Motivation, Engagement, and Thriving in User Experience), this study validated five Technology-based Experience of Need Satisfaction (TENS) scales (Adoption, Interface, Task, Behavior, and Life) among Chinese university students. In Phase I, 320 students completed the translated TENS scales. Three scales demonstrated acceptable reliability and model fit, while the TENS-Interface and TENS-Task scales showed poor performance, particularly in dimensions that combined positively and negatively worded items. In Phase II, revised versions of these two scales were administered to a new sample (*N* = 189), with mixed-directional items reworded into a consistent positive format. CFA results indicated substantial improvements in model fit, internal consistency, and convergent validity. The findings underscore the importance of item wording consistency and the value of iterative validation when adapting instruments across cultural contexts. Importantly, this study extends the applicability of the METUX framework to Chinese higher education, offering empirical evidence that its core constructs are transferable when appropriate linguistic and contextual modifications are made. The refined TENS scales provide a robust foundation for assessing students’ basic psychological need satisfaction in AI-supported learning environments and offer methodological guidance for future scale adaptation in non-English-speaking contexts.

## 1 Introduction

Artificial Intelligence (AI) is rapidly transforming various sectors, from healthcare to finance, and is shaping how we live, work, and interact with the world (Rizvi et al., 2023). Within education, AI is not merely a technological add-on, but a catalyst for reshaping learning methodologies and educational frameworks (Heeg and Avraamidou, 2023; Zheng and He, 2021). This research adopts a broad view of AI, encompassing a range of technologies that aim to augment or supplement human intelligence (Holmes and Tuomi, 2022). While the potential benefits of AI in education, such as personalized learning, improved access to resources, and enhanced engagement, have been confirmed, the challenges presented by AI must also be addressed (Abulibdeh et al., 2025). As recent reviews of the literature have shown, AI raises concerns regarding digital fatigue, loneliness, technostress, and reduced face-to-face interactions, leading to social isolation and anxiety (Klimova and Pikhart, 2025; Zhai et al., 2021). Therefore, it is crucial to assess the effect of AI technologies on students’ psychological needs and wellbeing.

One widely used tool to assess the effect of technologies on students’ psychological needs is the METUX (Motivation, Engagement and Thriving in User Experience) model (Peters et al., 2018). It is grounded in Self-Determination Theory (SDT), identifying three basic psychological needs: autonomy, competence, and relatedness (Ryan and Deci, 2000). This model posits that evaluating technology is intrinsically linked to psychological need satisfaction across diverse spheres of user experience (Burnell et al., 2023; Peters et al., 2018), offering a structured approach to understanding AI-based need satisfaction.

The METUX model is typically evaluated through the Technology-based Experience of Need Satisfaction (TENS) scales, developed by Peters et al. (2018). These scales evaluate need satisfaction caused by a specific technology across six spheres of experience: adoption, interface, task, behavior, life, and society. In 2021, the TENS-Interface and TENS-Task scales were applied and validated in higher education settings to assess students’ need satisfaction while using digital learning tools (Jeno et al., 2021). Subsequently, in 2023, four TENS scales (interface, task, behavior, and life) were applied to Facebook, TikTok, Blackboard, and Moodle, demonstrating good psychometric properties across these four technologies (Burnell et al., 2023).

However, to date, no study has investigated how AI technologies shape students’ psychological needs. This gap is particularly important because AI-supported learning environments differ in several ways from the digital technologies previously examined within the METUX framework. Earlier validation studies primarily focused on learning management systems and social media platforms, which mainly support content delivery, communication, or information access (Burnell et al., 2023; Jeno et al., 2021). In contrast, contemporary AI tools can generate responses, provide personalized feedback, engage in conversational interactions, and adapt dynamically to users’ inputs (Abulibdeh et al., 2025; Holmes and Tuomi, 2022). These characteristics may shape students’ experiences of autonomy and competence, and may also support relatedness in more socially interactive AI contexts (Crawford et al., 2024). Therefore, it cannot be assumed that psychometric properties established for conventional digital technologies will automatically generalize to AI-supported learning environments. Furthermore, while validation studies of the TENS scales have been conducted in English-speaking contexts (Burnell et al., 2023; Jeno et al., 2021), the psychometric properties of the TENS scales have yet to be tested in Chinese educational settings specifically in the context of AI technologies. As such, there is limited evidence regarding the model’s applicability in non-English-speaking cultural contexts when evaluating AI technologies in higher education. Given that China has a vast higher education system with numerous universities and students increasingly utilizing AI (Wan, 2021), understanding the psychometric properties of these scales within this context is of paramount importance for ensuring accurate and culturally relevant assessments of students’ experiences with AI technologies.

This study addresses this important gap by translating and validating the TENS scales among Chinese university students in the context of AI technologies. In the present study, AI technologies primarily refer to generative AI tools commonly used by Chinese university students in higher education contexts, such as DeepSeek, Doubao, and Kimi. These tools may be used for learning support, information retrieval, content generation, and broader daily-life assistance, depending on the specific METUX sphere being assessed. The TENS scales consist of five components: four developed by Peters et al. (2018), namely the Autonomy and Competence in Technology Adoption scale (ACTA), the TENS-Interface, the TENS-Task, and the TENS-Life scales, along with the TENS-Behavior scale from Burnell et al. (2023). This validation process, focusing on cultural adaptation, factor structure, and internal reliability, provides a foundation for analyzing how AI technologies shape student experience in Chinese higher education and lays the groundwork for future studies linking need satisfaction to key outcomes, such as engagement and wellbeing.

## 2 Theoretical framework

The METUX model assesses how three basic psychological needs are supported or frustrated by technology use across six distinct spheres (Burnell et al., 2023; Peters et al., 2018):

### 2.1 Adoption

The decision-making experience between becoming aware of a new technology and acquiring it. In this research it refers to students’ motivation for starting to use AI technologies.

### 2.2 Interface

The experience of interacting with a technology via its interface during use. In this research it refers to students’ perceptions of the AI technology’s usability.

### 2.3 Task

The experience of engaging in a technology-specific task. In this research it refers to students’ experience while using the AI technology to get study ideas or solve problems.

### 2.4 Behavior

The experience of engaging in a behavior (that a technology is intended to support). In this research it refers to students’ experiences while using the AI technology in learning process.

### 2.5 Life

An individual’s overall experience of life including all that is outside or beyond the technology. In this research it refers to broader impacts of the AI technology on students’ daily life.

### 2.6 Society

The experiences of all members of a society beyond the users of a technology. This dimension was not included in the present study. As Peters et al. (2018) stated “*this level of societal impact requires the consideration of interdependent factors, and therefore, will be, by far, the most difficult to accurately assess and will require multidisciplinary collaboration and new methods.*” While we acknowledge the importance of the societal dimension and its potential impact on the broader community, this study is primarily focused on understanding individual student experience and the psychological impact of AI technologies on their needs within the higher education environment. A comprehensive analysis of the societal impact of AI in education is thus beyond the scope and resources of the current research, as it would necessitate different methodologies, such as large-scale surveys, policy analysis, or qualitative studies involving diverse stakeholders beyond the student population. We recognize that the societal impact of AI in education is a crucial area for future research, and we encourage researchers to build upon our findings by investigating these important societal dimensions. As such, the society dimension was excluded from the current validation effort.

## 3 Phase I: initial validation of the scales

### 3.1 Method

#### 3.1.1 Participants

A convenience sampling approach was adopted for participant recruitment due to its practicality and cost-effectiveness in reaching university students (Creswell and Creswell, 2018). Participants completed the Chinese versions of the scales, reflecting on their experiences with generative AI tools commonly used by Chinese university students, such as DeepSeek, Doubao, and Kimi. A total of 320 survey responses were collected from three institutions across two provinces,^1^ comprising 156 males and 164 females. In the sample, 162 respondents were in their first year, 92 were in their second year, and 66 were in their third and final year. Institutional ethical approval was obtained (ref: SMEA250601) for all phases, and all methods adhered to relevant ethical guidelines and regulations.

#### 3.1.2 Instruments

This study employs the TENS scales, with five scales combined into a single questionnaire administered to participants, to assess the effects of AI technologies on students’ psychological needs. All scale items are rated on a 7-point Likert scale, ranging from 1 (strongly disagree) to 7 (strongly agree). Each dimension score is calculated by averaging the items within that dimension, resulting in a range between 1 and 7. Sample items for each scale are displayed in Table 1 to demonstrate the differences between the spheres; the full scales can be found in Supplementary Appendices 1–5. The scales used are described below:

**TABLE 1:** Sample item from each scale.

Scale	Sample item
ACTA scale	I am required to use it (e.g., by my school, teacher, peers).
TENS-interface scale	The AI technology provides me with useful options and choices
TENS-task scale	It’s easy to use AI to generate study ideas or solve problems.
TENS-behavior scale	AI helps me feel confident in my ability to engage in my learning activities.
TENS-life scale	Using AI technology has made me feel less capable in my life.

##### 3.1.2.1 ACTA

This 14-item instrument measures students’ motivation for adopting AI technologies. The scale comprises five composite dimensions: intrinsic motivation, identified motivation, introjected motivation, external motivation, and competence.

##### 3.1.2.2 TENS-interface

This 10-item scale assesses the effects of AI technologies on the satisfaction of psychological needs during AI use. The scale comprises two composite dimensions: autonomy and competence.

##### 3.1.2.3 TENS-task

This 8-item scale measures the effects of AI technologies on the satisfaction of psychological needs related to generating study ideas or solving problems. The scale comprises two composite dimensions: autonomy and competence.

##### 3.1.2.4 TENS-behavior

This 6-item scale measures the effects of AI technologies on the satisfaction of psychological needs related to engagement in learning activities. The scale comprises two composite dimensions: autonomy and competence.

##### 3.1.2.5 TENS-life

This 10-item instrument measures the broad effects of AI technologies on the satisfaction of psychological needs in students’ daily life. This scale includes three composite dimensions: autonomy, competence, and relatedness.

Relatedness is essential to overall wellbeing within SDT, but its inclusion in each METUX sphere depends on the nature of the technology, the sphere being assessed, and whether respondents can meaningfully interpret the relatedness items in that context. In developing the present questionnaire, we referred to both Peters et al.’s (2018) original TENS instruments and Burnell et al.’s (2023) later refinement of the METUX scales. In Peters et al.’s (2018) original instruments, relatedness items were included as optional items in the TENS-Interface and TENS-Task scales. In Burnell et al.’s (2023) refined scales, relatedness was excluded from the Interface scale, while relatedness items were retained in the Task, Behavior, and Life scales.

In the present study, relatedness was retained in the TENS-Life scale but was not included in the Interface, Task, and Behavior spheres. This decision was based primarily on pilot-testing evidence. During cognitive interviews, participants reported difficulty understanding how items concerning interpersonal closeness, belonging, meaningful connection, or relationships with others applied to their use of AI tools such as Doubao, DeepSeek, and Kimi, especially when these tools were used for individual learning support, information retrieval, content generation, or daily-life assistance. Although conversational AI tools may potentially produce relational or socially meaningful experiences, particularly when users anthropomorphize the technology or develop a sense of connection with conversational agents (Airenti, 2015; Christoforakos et al., 2021), the participants in this pilot study did not clearly interpret their AI use in terms of relatedness as framed by the existing TENS items. Therefore, to ensure construct clarity and data quality, the relatedness items were not included in these spheres. This decision was based on both the structure of prior METUX scale versions and pilot-testing evidence, rather than psychometric performance alone.

#### 3.1.3 Procedure

This study involved translating the TENS scales into Chinese and subsequently evaluating their psychometric properties. To ensure the linguistic and conceptual equivalence of the original English version of the questionnaire in the Chinese context, a multi-step translation process was conducted following the International Test Commission guidelines (Tanzer and Sim, 1999). First, two bilingual translators whose native language is Chinese independently performed the forward translation of the questionnaire from English to Chinese. Their two translated versions were then reviewed and reconciled by a third researcher with expertise in psychology and education, resulting in a single integrated Chinese version.

Next, two independent bilingual translators, blind to the original English version, performed a back translation from Chinese to English. This procedure was implemented to identify any semantic discrepancies and ensure the conceptual equivalence of the translated items (Klotz et al., 2023). The back-translated version was then rigorously compared with the original English version, and minor adjustments were made to the Chinese text as necessary to optimize fidelity. For example, the original item “I will feel bad about myself if I didn’t try it” was adapted to “I will feel bad about myself (ashamed) if I didn’t try it, because I feel I should be using the latest AI technology.” This adaption was prompted by the item’s intention to measure introjected motivation, a construct characterized by internal pressures and feelings of obligation that drive behavior to avoid negative self-evaluations (Deci and Ryan, 2008). The back-translation process served to confirm that this adaptation retained the core element of negative self-evaluation associated with non-engagement with AI, while more explicitly capturing the dimension of internalized pressure related to perceived expectations around AI adoption.

Subsequently, a panel of three experts—specializing in psychology, education, and psychometric assessment, respectively—evaluated the translated items for clarity, cultural relevance, and conceptual consistency (Klotz et al., 2023). This multidisciplinary approach was crucial to ensuring the adapted instrument’s validity across different domains of expertise. Based on the experts’ feedback, minor revisions were implemented to enhance item comprehension and ensure a robust alignment with the intended constructs. For example, while a direct translation of “guilty” (内疚) exists in Chinese, the panel determined that the term does not carry the same cultural weight or emotional resonance as it does in English-speaking contexts. Therefore, it was adapted to “uneasy” (不安) to better resonate with the target population and more accurately capture the intended psychological state.

Finally, the preliminary Chinese version of the questionnaire was pilot tested with a sample of eight undergraduate students from Chinese universities. Cognitive interviews were conducted to assess their understanding of each item and identify any potentially confusing or ambiguous wording (Xu et al., 2023). During these interviews, students were specifically asked to verbalize their thought processes while answering the questions, allowing for a detailed understanding of how they interpreted each item in relation to their experiences with AI technologies. For example, most students reported a lack of interpersonal closeness when using AI, a finding that aligns with concerns about the potential for technology to create social isolation (Crawford et al., 2024). Based on the insights gained from the pilot study, the final version of the Chinese questionnaire was refined to ensure clarity, relevance, and cultural appropriateness for the target population.

#### 3.1.4 Data analysis

**FIGURE 1:**
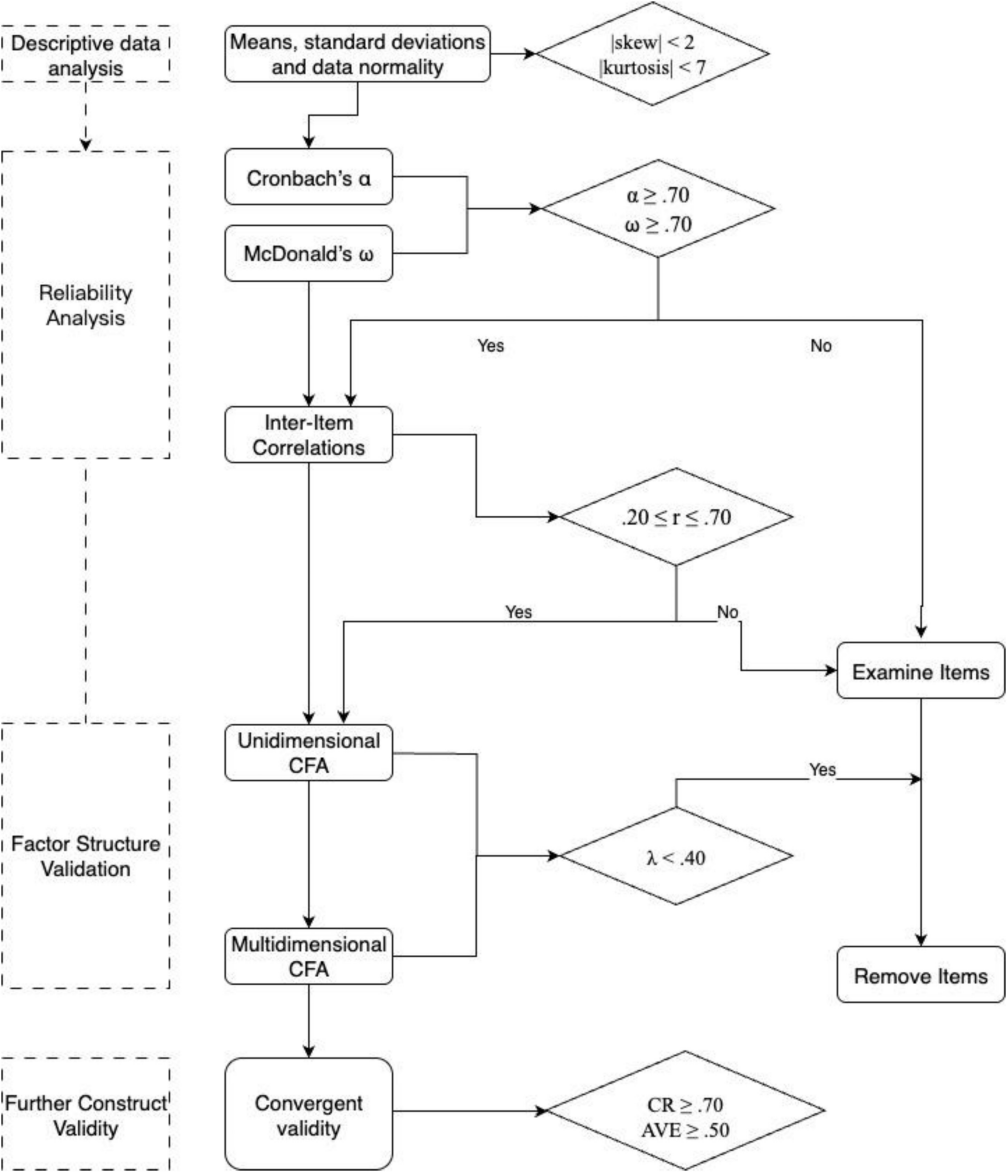
Stepwise flow of psychometric validation procedures. The diagram illustrates key steps for reliability and validity assessment, including internal consistency checks, inter-item correlations, CFA, and evaluation of convergent validity.

To examine the psychometric properties of the Chinese version of the TENS scales, the data analysis process included the following steps (see Figure 1). All analyses were conducted using SPSS 26.0 and Amos 24.0. Before conducting the psychometric analyses, all negatively worded items were reverse-scored so that higher scores consistently indicated higher levels of psychological need satisfaction within each dimension.

Descriptive statistics (means, standard deviations, skewness, and kurtosis) were calculated to assess the normality of item distributions.

Internal consistency reliability of each dimension was evaluated using Cronbach’s α, McDonald’s ω, and inter-item correlations. Cronbach’s α was used for comparability with prior research (Adamson and Prion, 2013), while McDonald’s ω was included due to its relaxed assumption of tau-equivalence and its ability to more accurately reflect true reliability in the presence of heterogeneous factor loadings (Dunn et al., 2014). Inter-item correlations help ensure that translated items within each dimension are behaving consistently in this new linguistic and cultural context (Piedmont and Hyland, 1993).

Confirmatory Factor Analysis (CFA) was performed in two sequential steps to validate the hypothesized multidimensional structure of each scale, which was derived from the METUX model. First, individual CFAs were conducted for each dimension separately to examine unidimensional factor structures. These were followed by integrated CFAs in which all dimensions within each scale were modeled concurrently, enabling a comprehensive evaluation of the full-scale structure. Model fit was evaluated using multiple indices, including the Comparative Fit Index (CFI), Tucker–Lewis Index (TLI), Root Mean Square Error of Approximation (RMSEA), and Standardized Root Mean Square Residual (SRMR). Values of CFI and TLI above 0.90, RMSEA below 0.08, and SRMR below 0.08 were considered indicative of acceptable model fit (Hu and Bentler, 1999). Furthermore, standardized factor loadings (λ) were examined for each item to assess the strength of its relationship with the corresponding latent construct. Following recommendations by Hair (2011), loadings above 0.50 were considered acceptable, while items below 0.40 were considered for deletion.

Following CFA, additional psychometric indices were computed to further evaluate the construct validity of the scales. Composite Reliability (CR) was used to assess the internal consistency of each latent construct, offering a more precise estimate than Cronbach’s α by incorporating factor loadings. Average Variance Extracted (AVE) was calculated to evaluate convergent validity, indicating the extent to which a construct explains the variance of its items (Fornell and Larcker, 1981; Hair, 2011). These indices provide evidence for the construct validity of the scales by assessing both the internal consistency and explanatory power of each latent factor.

### 3.2 Results

#### 3.2.1 Descriptive results

Means, standard deviations and data normality (skewness and kurtosis) are presented in Table 2. Skewness values ranged from –0.75 to 0.37 and kurtosis values ranged from –0.15 to 2.61. All values fell within acceptable thresholds for approximate normality (i.e., |skew| < 2; |kurtosis| < 7), as recommended by Kline (2023).

**TABLE 2:** Descriptive statistics for TENS scales in this study.

Scale	Dimension	Item label	Mean	Standard deviation (SD)	Skewness	Kurtosis	Cronbach’s α	McDonald’s ω	Factor loading (λ)
ACTA	Intrinsic	ACTA2	4.53	1.10	–0.39	1.88	0.794	0.800	0.65
		ACTA7	4.73	1.10	–0.42	1.63			0.80
		ACTA10	4.42	1.17	–0.41	1.56			0.82
	Identified	ACTA3	4.84	1.22	–0.65	1.02	0.823	0.828	0.81
		ACTA4	4.79	1.20	–0.58	1.29			0.83
		ACTA9	4.73	1.10	–0.51	1.94			0.70
	Introjected	ACTA5	3.55	1.37	0.03	0.11	0.790	0.793	0.81
		ACTA6	3.39	1.28	–0.06	0.00			0.69
		ACTA12	3.75	1.32	–0.35	0.33			0.74
	External	ACTA1	3.92	1.46	–0.19	–0.15	0.716	0.763	0.34
		ACTA8	3.56	1.39	–0.04	0.05			0.92
		ACTA11	3.51	1.39	0.04	0.02			0.85
	Competence	ACTA13	4.51	1.09	–0.23	1.66	0.697	–	–
		ACTA14	4.48	1.10	–0.38	1.70			–
TENS-Interface	Competence	IF1	4.35	1.25	–0.40	0.59	0.452	0.521	0.14
		IF2	4.47	1.07	–0.20	1.84			0.27
		IF3	4.12	1.27	0.29	0.62			–0.81
		IF4	4.11	1.21	0.17	0.86			–0.71
		IF5	3.83	1.20	0.37	0.96			–0.69
	Autonomy	IF6	4.79	1.15	–0.57	1.55	0.611	0.684	0.67
		IF7	4.60	1.01	–0.26	1.95			0.99
		IF8	4.47	1.24	0.03	0.61			–0.11
		IF9	4.20	1.29	0.27	0.49			–0.21
		IF10	4.42	1.34	0.05	0.28			–0.12
TENS-task	Competence	TASK1	4.39	0.96	–0.15	2.61	0.02	0.098	0.78
		TASK2	4.65	1.19	–0.65	1.30			0.64
		TASK3	4.11	1.18	–0.01	0.74			–0.42
		TASK4	4.10	1.19	0.15	0.93			–0.32
	Autonomy	TASK5	4.32	1.23	0.10	0.64	0.877	0.879	0.88
		TASK6	4.25	1.30	0.21	0.38			0.77
		TASK7	4.44	1.33	0.03	0.33			0.85
		TASK8	4.26	1.27	0.20	0.56			0.71
TENS-behavior	Autonomy	BEHA1	4.96	1.22	–0.75	1.39	0.816	0.827	0.78
		BEHA2	4.53	1.06	–0.43	2.61			0.64
		BEHA3	4.73	1.09	–0.63	2.15			0.92
	Competence	BEHA4	4.52	1.08	–0.26	2.01	0.855	0.858	0.73
		BEHA5	4.73	1.11	–0.45	1.74			0.86
		BEHA6	4.75	1.10	–0.40	1.67			0.85
TENS-life	Autonomy	LIFE1	4.62	1.24	–0.27	0.58	0.767	0.779	0.42
		LIFE2	4.05	1.22	0.01	0.89			0.74
		LIFE3	4.09	1.23	0.28	0.72			0.76
		LIFE4	4.29	1.29	0.08	0.40			0.79
	Competence	LIFE5	4.24	1.28	–0.06	0.45	0.837	0.838	0.74
		LIFE6	4.13	1.27	0.23	0.55			0.85
		LIFE7	4.50	1.30	–0.10	0.30			0.80
	Relatedness	LIFE8	3.94	1.19	–0.44	1.31	0.784	0.784	0.73
		LIFE9	4.01	1.14	–0.22	1.84			0.79
		LIFE10	3.89	1.19	–0.26	1.14			0.70

#### 3.2.2 Reliability analysis: Cronbach’s α, McDonald’s ω and inter-item correlations

To evaluate the internal consistency and construct coherence of the five TENS scales, Cronbach’s α, McDonald’s ω and Pearson inter-item correlations were computed for each dimension of the scales.

##### 3.2.2.1 Cronbach’s α and McDonald’s ω

The reliability of the scales was assessed using both Cronbach’s α and McDonald’s ω, with results presented in the Table 2. Most dimensions demonstrated acceptable to high reliability, with α and ω values exceeding the commonly recommended threshold of 0.70 (Hair, 2011). For example, all dimensions within the ACTA scale showed α values ranging from 0.697 to 0.823 and ω values from 0.763 to 0.828, indicating good internal consistency. Similarly, the autonomy and competence dimensions of the TENS-Behavior and TENS-Life scales yielded strong reliability indices (e.g., TENS-Behavior Competence: α = 0.855, ω = 0.858).

However, some dimensions, particularly the competence dimensions in TENS-Interface (α = 0.452, ω = 0.521) and TENS-Task (α = 0.02, ω = 0.098), fell well below the acceptable range. The autonomy dimension of TENS-Interface also showed marginal reliability (α = 0.611, ω = 0.684). These unexpectedly low values may reflect conceptual inconsistencies among items or limited inter-item correlation. To further examine the internal structure and address the low reliability, inter-item correlations were subsequently conducted.

##### 3.2.2.2 Inter-item correlations

- ACTA Scale

Inter-item correlations were examined across the five dimensions of the ACTA scale: Intrinsic, Identified, Introjected, External, and Competence (see Supplementary Appendix 6). Overall, most dimensions demonstrated acceptable internal consistency, with item correlations ranging from 0.290 to 0.778. Although ACTA3 showed weak correlations with several items outside its dimension (e.g., ACTA5, ACTA11), it displayed strong correlations with items within the Identified regulation dimension (e.g., ACTA3–ACTA4 = 0.671; ACTA3–ACTA9 = 0.571), supporting its construct alignment. The primary criterion (*r* > 0.30) for item inclusion was adequate convergence within dimension items (Briggs and Cheek, 1986). These patterns were further evaluated through CFA.

- TENS-Interface Scale

Inter-item correlations for the TENS-Interface dimensions revealed notable inconsistencies (see Supplementary Appendix 7). Within the competence dimension (IF1–IF5), IF3 showed weak or negative correlations with other items (e.g., IF3–IF1 = –0.061; IF3–IF2 = –0.175), suggesting possible semantic reversal or misalignment. Similarly, in the autonomy dimension (IF6–IF10), IF7 displayed negative correlations with IF9 and IF10 (*r* = –0.206, –0.111), potentially indicating a directional inconsistency. These items were retained for further evaluation in CFA.

- TENS-Task Scale

Inter-item correlations were examined for the TENS-Task dimensions (see Supplementary Appendix 8). The autonomy dimension (T5–T8) showed strong internal consistency, with all item pairs demonstrating significant positive correlations ranging from 0.545 to 0.746. In contrast, the competence dimension (T1–T4) revealed problematic internal coherence: T1 and T2 showed negative correlations with T3 and T4 (e.g., T1–T3 = –0.284; T2–T4 = –0.092), suggesting possible semantic or directional inconsistency. These patterns were further examined through CFA.

- TENS-Behavior Scale

Inter-item correlations for the TENS-Behavior dimensions indicated strong internal consistency (see Supplementary Appendix 9). Within the autonomy dimension (B1–B3), item correlations ranged from 0.498 to 0.712. Similarly, the competence dimension (B4–B6) showed very strong inter-item correlations ranging from 0.622 to 0.734, suggesting excellent internal coherence. No item demonstrated low or inconsistent correlations, supporting the structural integrity of the dimensions.

- TENS-Life Scale

Inter-item correlations for the TENS-Life dimensions demonstrated acceptable to strong internal consistency (see Supplementary Appendix 10). Within the autonomy dimension (LIFE1–LIFE4), item correlations ranged from 0.267 to 0.624. Although LIFE1 showed slightly lower correlations with LIFE3 and LIFE4, all values were statistically significant. The Competence dimension (LIFE5–LIFE7) exhibited consistently strong correlations (*r* = 0.588– 0.679), and the Relatedness dimension (LIFE8–LIFE10) also showed high internal consistency (*r* = 0.511–0.577).

Taken all together, the inter-item correlations indicated that while several dimensions (e.g., autonomy in TENS-Task and TENS-Behavior) demonstrated strong and consistent item associations, others, most notably the competence dimensions in TENS-Interface and TENS-Task, exhibited weak or even negative correlations between items. These findings suggest uneven psychometric performance across dimensions and provided an empirical foundation for subsequent CFA and item-level model refinements. To further confirm the factor structure, a CFA was conducted, which is discussed in the next section.

#### 3.2.3 Factor structure validation: CFA by individual dimensions

##### 3.2.3.1 Model fit evaluation

Each dimension of the five TENS scales was subjected to a separate CFA to confirm its unidimensional structure and support construct validity prior to testing full models. The results (see Supplementary Appendix 11) indicated varied levels of model fit across dimensions. Some dimensions showed strong support on selected fit indices, such as the TENS-Task autonomy dimension (CFI = 1.00, RMSEA = 0.00, SRMR = 0.00). However, other dimensions displayed elevated RMSEA values despite acceptable or strong values on alternative indices, such as the TENS-Life autonomy dimension (CFI = 0.95, RMSEA = 0.18) and the TENS-Behavior autonomy dimension (CFI = 1.00, RMSEA = 0.62). These results should therefore be interpreted cautiously, particularly because RMSEA may perform poorly or be inflated in models with low degrees of freedom (Kenny et al., 2015). Rather than relying on any single fit index, model evaluation was based on the combined consideration of CFI, TLI, RMSEA, SRMR, item-level factor loadings, and reliability evidence.

Several dimensions exhibited clear evidence of poor model fit, notably the TENS-Interface autonomy dimension (CFI = 0.31, TLI = –0.38, RMSEA = 0.53), and TENS-Interface competence (CFI = 0.69, RMSEA = 0.29), both suggesting potential problems in the item structure. Similarly, TENS-Task competence (CFI = 0.80, RMSEA = 0.25) showed only marginal fit. These findings warranted further analysis including potential item revision and revalidation, particularly for the TENS-Interface and TENS-Task scales.

##### 3.2.3.2 Item-level loading evaluation

Across the ACTA scale, most items demonstrated satisfactory loadings above 0.60 (see Table 2), indicating acceptable item-construct alignment (Hair, 2011). However, ACTA1 (λ = 0.34) within the external dimension fell below the acceptable threshold (0.40), suggesting weak representation of the intended factor and requiring further attention.

For the TENS-Interface scale, the results revealed substantial issues (see Table 2). Within the competence dimension, three items, namely, IF3 (λ = –0.81), IF4 (λ = –0.71), and IF5 (λ = -0.69), showed large negative loadings, while the other two items (IF1 and IF2) had weak positive loadings (λ = 0.14 and 0.27, respectively). Similarly, the autonomy dimension exhibited problematic indicators, with IF8, IF9, and IF10 showing negative loadings, and only IF6 and IF7 displaying acceptable values.

The TENS-Task scale also revealed a problematic competence dimension (see Table 2). TASK3 (λ = –0.42) and TASK4 (λ = –0.32) exhibited negative loadings, undermining their validity. In contrast, the autonomy dimension performed well, with all item loadings above 0.70.

Both TENS-Behavior and TENS-Life scales demonstrated overall strong and consistent factor loadings (see Table 2), supporting the structural integrity of their constructs. All dimensions within these two scales had loadings exceeding 0.60, except for LIFE1 (λ = 0.42), which was marginal but still within acceptable range for exploratory contexts.

In sum, the CFA results indicated that most dimensions across the five scales, the majority of items exhibited acceptable to strong factor loadings (λ ≥ 0.50). However, the TENS-Interface and TENS-Task scales require substantial revision due to multiple low or negative loadings. These issues appeared only in dimensions that mixed positively and negatively worded items, namely within autonomy and competence dimensions of the TENS-Interface and competence dimension of TENS-Task scales. Such inconsistency further supports the decision to revise the item phrasing for revalidation purposes of TENS-Interface and TENS-Task scales (see Phase II for details).

## 4 Phase II: re-validation of TENS-interface and TENS-task scales

### 4.1 Rationale for revisions

Given the low internal consistency and poor model fit observed in the original TENS-Interface and TENS-Task dimensions, particularly those combining positively and negatively worded items, it was hypothesized that the mixed item directionality may have adversely affected the psychometric properties, especially among Chinese respondents. It should be noted that all negatively worded items were reverse-scored before the Phase I analyses. Therefore, the negative or inconsistent factor loadings observed in the TENS-Interface and TENS-Task scales did not reflect uncorrected item direction, but rather indicated that these reverse-worded items did not function psychometrically as intended in the Chinese AI-use context. Several studies have shown that negatively worded items can lead to lower internal consistency and poor model fit because they are often misinterpreted or processed differently than positively worded items (Harzing, 2006; Woods, 2006). This is of particular concern with Mandarin speakers, as research suggests that they may be more prone to acquiescence bias or semantic reversal errors when responding to negatively worded items due to linguistic and cultural factors (Wu, 2008; Zeng et al., 2020). Therefore, rather than advancing to full-scale CFA involving multiple dimensions simultaneously, a second phase of data collection was undertaken. In this phase, mixed positively and negatively worded items were reworded to maintain a consistent positive direction, and the revised scales were re-validated to examine their reliability and structural validity.

### 4.2 Methods

#### 4.2.1 Participants

A total of 189 undergraduate students (M_*age*_ = 19.89, SD = 1.27; 42 males and 147 females) were recruited from two institutions across two provinces^2^ to participate in Phase II of the study. These participants had not taken part in the first data collection, ensuring sample independence between phases. All participants provided informed consent prior to participation.

#### 4.2.2 Instruments

Phase II focused on re-evaluating two scales of the METUX model: the TENS-Interface and TENS-Task scales. Since it was hypothesized that the mixing of positively and negatively worded items within the same dimension may have contributed to the low reliability and poor factor structure observed in Phase I, only those dimensions that originally contained a mixture of positive and negative items were revised into a consistently positive format, aiming to reduce respondent confusion and improve psychometric clarity. Following the original TENS scoring framework (Peters et al., 2018), these negatively worded items were treated as reverse-scored indicators within need satisfaction dimensions rather than as separate measures of need frustration. Accordingly, the negatively worded items of the competence and autonomy dimensions of TENS-Interface scale and the competence dimension of TENS-Task scale were revised into a positive formulation. For example, “I found the interface and controls clear and easy to understand” was used instead of “I found the interface and controls confusing,” “It was easy to use AI technology” replaced “It wasn’t easy to use AI technology,” and “I find using AI to generate study ideas or solve problems too challenging” was revised as “I find using AI to generate study ideas or solve problems manageable and within my ability.” These revisions were designed to preserve the intended need-satisfaction dimension of each item while changing the wording direction to reduce reverse-wording-related method effects and respondent confusion. The revised scales can be found in Supplementary Appendices 12, 13, respectively. As the TENS-Task autonomy dimension contained only negatively worded items, it was left unchanged in Phase II to allow for meaningful comparison with the revised dimensions.

#### 4.2.3 Data analysis

The same analytical procedures as described in Phase I were applied in Phase II to examine the revised TENS-Interface and TENS-Task scales, including reliability testing, inter-item correlations, and CFA.

### 4.3 Results

#### 4.3.1 descriptive results

The skewness and kurtosis results suggested that the revised TENS-Interface and TENS-Task items demonstrated acceptable univariate normality (see Table 3). Most items showed near-symmetric distributions and appropriate levels of peakiness. Notably, the modified (positively worded) competence items exhibited more balanced distributional properties compared to their original counterparts, indicating improved item clarity and response consistency. Items within the TENS-Task autonomy dimension, which retained their original negatively worded format, continued to display slightly more variability, suggesting that item wording may influence response patterns.

**TABLE 3:** Descriptive statistics for revised TENS scales in this study.

Scale	Dimension	Item label	Mean	Standard deviation (SD)	Skewness	Kurtosis	Cronbach’s α	McDonald’s ω	Factor loading (λ)
TENS-interface (revised)	Competence	IF1	4.88	1.11	–0.19	0.61	0.875	0.875	0.737
		IF2	4.85	1.05	–0.09	0.59			0.677
		IF3	5.10	1.00	–0.23	1.37			0.795
		IF4	5.07	1.10	–0.31	0.47			0.804
		IF5	5.16	0.93	–0.22	0.48			0.813
	Autonomy	IF6	5.23	1.01	–0.07	–0.37	0.880	0.880	0.713
		IF7	4.83	0.94	0.23	0.22			0.836
		IF8	4.98	0.98	0.11	–0.14			0.774
		IF9	4.71	0.97	0.37	0.04			0.750
		IF10	4.89	0.97	0.19	–0.06			0.787
TENS-task (revised)	Competence	TASK1	4.87	1.04	0.09	–0.36	0.861	0.862	0.779
		TASK2	5.04	1.00	0.06	0.25			0.858
		TASK3	4.98	1.01	0.06	–0.05			0.797
		TASK4	4.79	1.06	0.02	0.46			0.692
	Autonomy	TASK5	4.01	1.20	0.33	0.07	0.629	0.667	0.909
		TASK6	4.65	1.21	–0.17	0.09			0.304
		TASK7	3.71	1.34	0.25	–0.07			0.577
		TASK8	3.80	1.26	0.08	0.31			0.475

#### 4.3.2 Reliability analysis

To evaluate the internal consistency of the revised scales, both Cronbach’s α and McDonald’s ω were computed for each dimension (see Table 3). The TENS-Interface scale showed excellent reliability for both competence (α = 0.875, ω = 0.875) and autonomy (α = 0.880, ω = 0.880) dimensions. Similarly, the TENS-Task scale demonstrated high reliability for the competence dimension. However, the autonomy dimension of the TENS-Task showed only acceptable reliability (α = 0.629, ω = 0.667), which is lower than desirable but still within the marginal threshold.

Inter-item correlation analysis generally supported the internal coherence of most dimensions within the revised scales. For both revised TENS-Interface and TENS-Task scales, most item pairs demonstrated moderate to strong positive correlations (*r* = 0.466–0.716) (see Supplementary Appendices 14, 15), indicating satisfactory homogeneity within dimensions (Briggs and Cheek, 1986). However, a few anomalies were observed within the autonomy dimension of the TENS-Task scale. For example, some items, particularly TASK6 and TASK8, exhibited relatively weak inter-item correlations (e.g., *r* = 0.087). These findings highlight the need for continued examination of specific items in light of their contributions to overall reliability and factor structure in subsequent CFA results.

Taken together, the psychometric quality of the revised scales improved substantially, particularly for those dimensions that previously combined both positively and negatively worded items. The revised versions, now uniformly worded in a positive direction, demonstrated notably higher internal consistency and more coherent item correlations, especially in the TENS-Interface and TENS-Task competence dimensions, where low reliability and problematic factor loadings were initially observed in Phase I.

#### 4.3.3 Factor structure validation: CFA by individual dimensions

The results indicated acceptable to excellent model fit across all dimensions (see Supplementary Appendix 16). Specifically, for the TENS-Interface competence dimension, although the RMSEA was slightly elevated, other indices supported an adequate fit (CFI = 0.94, GFI = 0.93, SRMR = 0.05). The TENS-Interface autonomy dimension demonstrated improved model fit (CFI = 0.98, TLI = 0.96, SRMR = 0.03). Regarding the TENS-Task scale, both competence and autonomy dimensions exhibited excellent model fit, with CFI and GFI values of 1.00 and SRMR values below 0.03, indicating strong structural validity of the revised scales.

In terms of standardized factor loadings (λ), most items in the revised scales exceeded the recommended threshold of 0.50 (see Table 3), indicating strong relationships with their respective latent constructs. For the TENS-Interface scale, all items loaded acceptably onto their intended dimensions, with competence items ranging from 0.677 to 0.813 and autonomy items from 0.713 to 0.836. Similarly, the TENS-Task competence items demonstrated robust loadings (λ = 0.692–0.858). However, in the autonomy dimension of TENS-Task, one item (TASK6) fell notably below the recommended level (λ = 0.304), suggesting it may not align well with the underlying construct. This will be further evaluated in the full-scale CFA to determine whether item removal is warranted.

Following the confirmation of improved model fit and acceptable item-level properties in the revised dimensions, integrated CFA was conducted for each full TENS scale. This aimed to validate the hypothesized multidimensional factor structures by simultaneously modeling all dimensions within each scale, thereby providing a more holistic assessment of structural validity.

## 5 Integrated validation: CFA of full scales

Following the prior step in which each dimension was individually examined through CFA, this section presents the results of integrated CFAs for five TENS scales (see Table 4). Namely, all dimensions within each scale were modeled simultaneously to evaluate whether the intended multidimensional structure was supported when assessed as a whole. Both original and revised versions of the scales were evaluated, and core indicators, including model fit indices, standardized factor loadings (λ), Composite Reliability (CR), and Average Variance Extracted (AVE), were systematically examined (see Table 5). Particular attention was paid to items with low factor loadings and their influence on overall model quality.

**TABLE 4:** CFA fit indices for the five TENS scales.

Model	χ ^2^	df	χ ^2^/df	CFI	TLI	RMSEA	SRMR	GFI	ECVI
ACTA	230.310	65.000	3.540	0.932	0.900	0.090	0.090	0.912	–
TENS-interface (original)	645.440	34.000	18.980	0.600	0.460	0.240	0.200	0.670	2.160
TENS-interface (revised)	77.190	33.000	2.339	0.961	0.947	0.084	0.042	0.916	0.645
TENS-task (original)	138.930	19.000	7.310	0.890	0.830	0.140	0.090	0.900	0.540
TENS-task (revised)	57.190	19.000	3.010	0.930	0.890	0.100	0.100	0.940	0.490
TENS-task (revised+TASK6 deletion)	18.963	13.000	1.459	0.987	0.979	0.049	0.038	0.973	0.260
TENS-behavior	67.110	8.000	8.390	0.950	0.900	0.150	0.050	0.930	–
TENS-life	120.580	32.000	3.770	0.940	0.920	0.093	0.047	0.930	0.522
TENS-life (LIFE1 deletion)	80.293	24.000	3.346	0.962	0.943	0.086	0.039	0.950	0.383

**TABLE 5:** Factor loadings and psychometric properties of TENS scales.

Scale	Dimension	Item label	Factor loading (λ)	Composite reliability (CR)	Average variance extracted (AVE)
ACTA	Intrinsic	ACTA2	0.73	0.825	0.613
		ACTA7	0.88		
		ACTA10	0.73		
	Identified	ACTA3	0.69	0.783	0.549
		ACTA4	0.67		
		ACTA9	0.85		
	Introjected	ACTA5	0.81	0.792	0.561
		ACTA6	0.67		
		ACTA12	0.76		
	External	ACTA1	0.38	0.778	0.565
		ACTA8	0.90		
		ACTA11	0.86		
	Competence	ACTA13	0.69	0.702	0.542
		ACTA14	0.78		
TENS-interface (revised)	Competence	IF1	0.78	0.871	0.576
		IF2	0.71		
		IF3	0.75		
		IF4	0.75		
		IF5	0.80		
	Autonomy	IF6	0.74	0.882	0.600
		IF7	0.81		
		IF8	0.79		
		IF9	0.75		
		IF10	0.78		
TENS-task (revised)	Competence	TASK1	0.79	0.867	0.620
		TASK2	0.87		
		TASK3	0.79		
		TASK4	0.69		
	Autonomy	TASK5	0.84	0.675	0.363
		TASK6	0.35		
		TASK7	0.61		
		TASK8	0.50		
TENS-task (revised+TASK6 deletion)	Competence	TASK1	0.79	0.864	0.616
		TASK2	0.86		
		TASK3	0.79		
		TASK4	0.69		
	Autonomy	TASK5	0.85	0.702	0.452
		TASK7	0.61		
		TASK8	0.51		
TENS-behavior	Autonomy	BEHA1	0.75	0.824	0.610
		BEHA2	0.75		
		BEHA3	0.84		
	Competence	BEHA4	0.71	0.859	0.673
		BEHA5	0.87		
		BEHA6	0.87		
TENS-life	Autonomy	LIFE1	0.38	0.770	0.472
		LIFE2	0.71		
		LIFE3	0.72		
		LIFE4	0.85		
	Competence	LIFE5	0.78	0.836	0.630
		LIFE6	0.79		
		LIFE7	0.81		
	Relatedness	LIFE8	0.78	0.781	0.544
		LIFE9	0.75		
		LIFE10	0.68		
TENS-life (LIFE1 deletion)	Autonomy	LIFE2	0.70	0.806	0.583
		LIFE3	0.72		
		LIFE4	0.86		
	Competence	LIFE5	0.78	0.836	0.630
		LIFE6	0.79		
		LIFE7	0.81		
	Relatedness	LIFE8	0.78	0.785	0.549
		LIFE9	0.76		
		LIFE10	0.68		

### 5.1 ACTA scale

The ACTA scale demonstrated acceptable overall model fit (e.g., CFI = 0.932, TLI = 0.900, RMSEA = 0.090, SRMR = 0.090), and all dimensions showed satisfactory psychometric performance (see Table 4). Most item loadings exceeded 0.67, and CR values ranged from 0.702 to 0.825. All AVE values surpassed the 0.50 threshold, indicating adequate convergent validity (see Table 5). Notably, one item (ACTA1) in the external motivation dimension had a factor loading of 0.38. Given that this dimension contained only three items, and the overall model fit remained acceptable, the item was retained to preserve structural integrity.

### 5.2 TENS-interface scale: original and revised

As can be seen in Table 4, the original version of the TENS-Interface scale exhibited poor model fit (e.g., CFI = 0.60, RMSEA = 0.24, SRMR = 0.20), suggesting substantial structural deficiencies. The Expected Cross-Validation Index (ECVI) for this model was 2.160, further suggesting poor replicability. To address these issues, all items were rephrased into a consistent positive format, thereby eliminating mixed directional wording. This revision substantially improved all model fit indices (e.g., CFI = 0.961, TLI = 0.947, RMSEA = 0.084, SRMR = 0.042) and internal consistency (CR > 0.87 for both dimensions), alongside a markedly reduced ECVI of 0.645, indicating enhanced generalizability and model parsimony. Psychometrically, the revised scale showed excellent internal consistency (CR = 0.871–0.882) and convergent validity (AVE = 0.576–0.600). Factor loadings for all items ranged from 0.71 to 0.81 (see Table 5), demonstrating strong alignment with the underlying constructs and reinforcing the structural integrity of the revised TENS-Interface scale.

### 5.3 TENS-task scale: three iterations of model development

The original TENS-Task scale exhibited marginal model fit (see Table 4), coupled with poor reliability and problematic inter-item correlations, particularly in the competence dimension that includes a mix of positively and negatively worded items, as identified in Phase I (see Table 2). To address these issues, all mixed-valence items in the competence dimension were reworded to adopt a consistent positive phrasing. In contrast, the autonomy dimension retained its original item wording to allow for direct comparison. The revised version of the scale was then re-evaluated using newly collected data in Phase II.

The revised version showed improved model fit (see Table 4), though the ECVI remained moderate at 0.49. While composite reliability (see Table 5) for the competence dimension was strong (CR = 0.867; AVE = 0.620), the autonomy dimension continued to show weak convergent validity (CR = 0.675; AVE = 0.363), with one item’s factor loading remaining below 0.40 (λ = 0.35).

To further test whether model refinement could enhance validity, a final model was tested with the problematic item (TASK6) removed. This version (see Table 4) yielded excellent fit indices (χ^2^/df = 1.46, CFI = 0.987, TLI = 0.979, RMSEA = 0.049, SRMR = 0.038), and the ECVI dropped significantly to 0.260—demonstrating a substantial improvement in model quality and cross-validation potential. Despite the minor reduction in item count, both internal consistency and AVE improved for the autonomy dimension (CR = 0.702, AVE = 0.452), supporting the decision to exclude the problematic item in the final validated version.

### 5.4 TENS-behavior scale

The TENS-Behavior scale exhibited generally good model fit (see Table 4), although the RMSEA value was somewhat elevated (0.15). As Kenny et al. (2015) highlighted, RMSEA is particularly sensitive to models with low degrees of freedom, which can lead to artificially inflated estimates and potentially misleading conclusions about model fit. In such cases, RMSEA alone may not accurately represent the model’s adequacy. Given that all other fit indices (e.g., CFI and SRMR) were within acceptable thresholds, the overall fit of the TENS-Behavior scale can still be considered satisfactory. Furthermore, the factor loadings were consistently strong (λ = 0.71–0.87), with CR values exceeding 0.82 and AVE values above 0.61, indicating robust internal consistency and convergent validity.

### 5.5 TENS-life scale

For the TENS-Life scale, one item (LIFE1) in the autonomy dimension showed a low factor loading (λ = 0.38). The AVE for this dimension also fell below the commonly recommended threshold of 0.50 (Fornell and Larcker, 1981). After removing LIFE1, the overall model fit improved notably (CFI increased from 0.94 to 0.962; RMSEA decreased from 0.093 to 0.086). In addition, CR and AVE also increased to 0.806 and 0.583, respectively. These results support the selective removal of low-loading items in cases where adequate redundancy exists within the scale.

**FIGURE 2:**
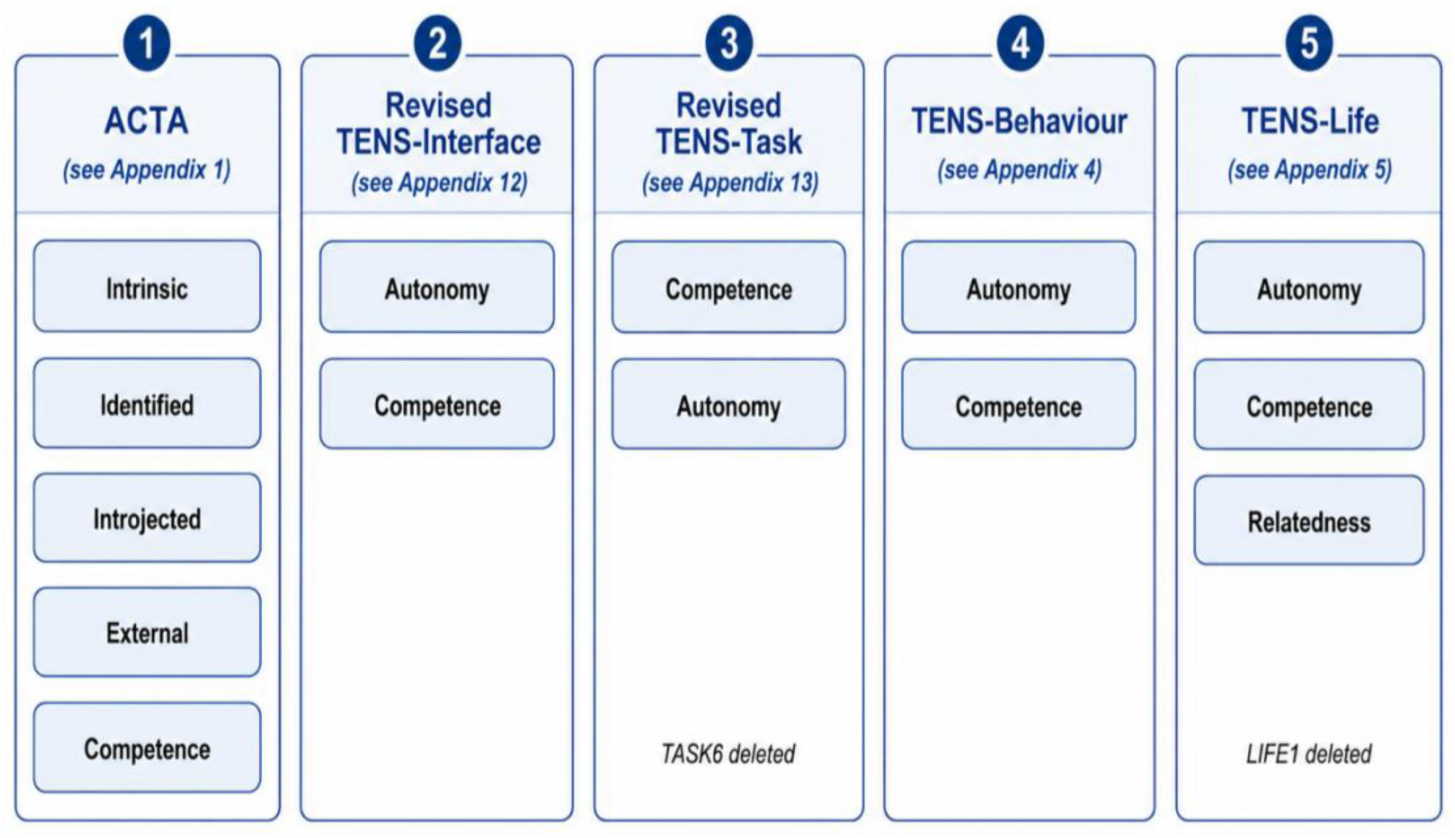
Final validated factor structure of the adapted TENS scales in the present study.

Collectively, these findings demonstrate that the revised TENS-Interface and TENS-Task scales offer significantly improved model fit and psychometric integrity compared to their original counterparts. The strategic removal of poorly performing items, particularly when combined with item rewording to reduce wording-related method effects, proved effective in optimizing scale validity. Nonetheless, caution was exercised in deleting items from dimensions with limited indicators to avoid compromising construct coverage. These findings underscore the need for careful validation and cultural refinement when adapting established scales for use outside their original Western development context. The final validated factor structure of the adapted TENS scales is summarized in Figure 2.

## 6 Discussion, implication, and limitation

The purpose of the present study was to validate the five TENS scales derived from the METUX model within the context of Chinese higher education. Through a two-phase validation, the findings supported the structural validity of the original ACTA, TENS-Behavior, and TENS-Life scales, while demonstrating significant improvements in the revised TENS-Interface and TENS-Task scales. These results provide preliminary evidence for the cultural transferability of the METUX framework and underscore the importance of context-sensitive instrument adaptation.

### 6.1 The impact of item wording consistency

One of the key findings of this study concerns the influence of mixed item wording on psychometric quality. In line with earlier warnings (Venta et al., 2022; Zeng et al., 2020), this study found that combining positively and negatively worded items within the same dimension (i.e., competence and autonomy dimensions in the TENS-Interface and competence dimension in the TENS-Task scales) was associated with poor model fit and low reliability. After rewording items into a consistent positive format, significant improvements were observed across all validation indices.

This finding should be interpreted primarily as evidence of wording-related method effects in cross-cultural scale adaptation. Although negatively worded items are sometimes included to reduce acquiescence bias (da Silva et al., 2022), previous studies have shown that they may introduce semantic reversal errors (DiStefano and Motl, 2006), cognitive burden, unintended method factors (Zeng et al., 2020), or careless responding (DiStefano and Motl, 2006; Woods, 2006), particularly when instruments are translated across linguistic and cultural contexts (Venta et al., 2022; Wu, 2008; Zhu et al., 2025). In the present study, the poor performance of the mixed-worded dimensions suggests that negatively worded reverse-scored items may have been processed differently from positively worded items by Chinese respondents, thereby weakening the structural coherence of the need satisfaction dimensions.

Importantly, the item revisions in Phase II were not intended to validate need frustration as a separate construct. Rather, consistent with the original TENS scoring framework, the negatively worded items were treated as reverse-scored indicators within need satisfaction dimensions (Peters et al., 2018). Rewording them into positively phrased items was therefore a pragmatic psychometric adaptation aimed at improving the clarity and structural validity of the AI-based need satisfaction scales in the Chinese higher education context. Future studies that aim to assess need frustration directly should include theoretically specified frustration items and examine their factor structure separately from need satisfaction.

### 6.2 Evaluating construct validity in Chinese higher education

The present study offers important insights into the cultural applicability of the METUX model and its associated scales. While the original TENS scales were developed and validated primarily in Western higher education settings (Burnell et al., 2023; Jeno et al., 2021), the current findings indicate that certain psychometric inconsistencies emerged when applying the scales to a Chinese university sample, particularly in dimensions that originally included mixed positively and negatively worded items.

Importantly, these inconsistencies do not appear to stem from fundamental differences in how core constructs such as autonomy or competence are experienced across cultures. For example, other TENS scales that measured autonomy using consistently worded items demonstrated strong structural validity and internal consistency. Rather, the issues observed in scales such as TENS-Interface and TENS-Task are more likely attributable to the cognitive burden or confusion introduced by item directionality, which may be amplified in non-native English contexts or among student populations less familiar with psychometric self-report measures. These findings underscore the importance of considering measurement artifacts, such as wording effects, when validating theoretically grounded scales in new cultural or linguistic contexts.

### 6.3 Theoretical and practical implications

The findings of this study yield both theoretical insights and practical guidance for researchers, educators, and practitioners working with the METUX model in diverse educational settings. As previously discussed, this study underscores a key theoretical insight: the consistency of item wording is not merely a methodological concern but a crucial factor influencing the construct validity of psychological scales, particularly in cross-cultural applications.

More specifically, the findings suggest that item wording may interact with cultural and cognitive response tendencies in cross-cultural educational research. Previous studies have indicated that respondents from different linguistic and cultural backgrounds may vary in their susceptibility to acquiescence bias, indirect response styles, and difficulties in processing negatively worded items (Wu, 2008; Zeng et al., 2020). In this study, the substantial improvement observed after rewording negatively phrased items suggests that such wording may impose additional cognitive burden or introduce response-pattern artifacts when psychological instruments are adapted for Chinese higher education contexts. Therefore, researchers should carefully evaluate negatively worded items during cross-cultural scale adaptation rather than assuming that item formats validated in the original language will function equivalently in new contexts.

Practically, the validated scales offer reliable tools for evaluating learner experiences in emerging AI-enabled educational environments. Given the specific cultural and educational context of this study, it is important to note that these scales have been validated for use with Chinese students. As AI technologies become increasingly integrated into teaching and learning processes within China, reliable assessment of students’ psychological needs, such as autonomy and competence, remains essential for optimizing engagement and wellbeing in digitally mediated contexts. While these scales demonstrate strong psychometric properties within the Chinese context, further validation would be necessary before using them in other countries or cultural settings. Researchers aiming to adapt or use these scales in different contexts can apply the rigorous validation process employed in this study, including item rewording, CFA, and assessment of CR and AVE, to ensure the scales’ reliability and validity in their target populations.

Moreover, the study underscores the necessity of iterative validation when adapting instruments across cultural boundaries. Scales developed in one linguistic or cultural context may not exhibit equivalent performance when applied elsewhere (He and van de Vijver, 2012). This study highlights the importance of conducting systematic pre-testing, reliability diagnostics, and CFA during the adaptation process rather than relying solely on translation and back-translation. Such procedures help to preserve conceptual fidelity while ensuring psychometric adequacy.

Finally, the findings raise important methodological considerations regarding item deletion. While removing low-loading items may enhance model fit, doing so may reduce the breadth and depth of construct representation, particularly when dimensions consist of only a few items (Hair et al., 2009). For example, ACTA1 in the external motivation dimension showed low factor loading, but was retained initially due to the limited number of items. This highlights the need to weigh statistical indicators against theoretical coverage, possibly incorporating expert review or qualitative feedback to ensure robust construct representation (DeVellis and Thorpe, 2021).

### 6.4 Limitations

Despite its contributions to the validation of the METUX model and its associated scales in a Chinese higher education context, this study is subject to several limitations that warrant consideration. First, although the study employed rigorous validation procedures, including inter-item correlation analysis, CFA, and iterative item revision, only self-reported measures were used. In addition, the cross-sectional design did not allow us to examine the temporal stability or test–retest reliability of the scales. This reliance on self-report data may introduce social desirability or common method bias, which can affect the accuracy of students’ perceived experiences, especially in relation to constructs like autonomy and competence. Mixed-method approaches incorporating interviews, behavioral measures, longitudinal designs, or test–retest procedures could provide richer insights, triangulate the findings, and further examine the temporal stability of the scales.

Second, although the study focused primarily on generative AI tools commonly used by Chinese university students, it did not distinguish between specific AI systems, functions, or usage scenarios in the validation analyses. These tools may be used across different METUX spheres, including adoption, interface interaction, learning-related tasks, learning engagement, and broader daily-life experiences. Treating them as a general category of generative AI technologies may therefore obscure potential differences in students’ psychological need satisfaction across different types of AI use. Moreover, other AI applications, such as intelligent tutoring systems, recommendation systems, or discipline-specific AI tools, may produce different patterns of autonomy, competence, and relatedness experiences. Future validation efforts should examine whether the TENS scales function equivalently across different AI systems, functions, and usage contexts, and whether additional constructs or item refinements are needed to reflect these emerging AI-supported environments.

A further limitation concerns the interpretation of negatively worded, reverse-scored items in the original TENS-Interface and TENS-Task scales. Although these items were treated in the present study as indicators within the need satisfaction framework, their negative phrasing may conceptually overlap with low need satisfaction, need-related difficulty, or need-undermining experiences. Rewording these items into consistently positive statements substantially improved model fit, reliability, and convergent validity, but it may also have narrowed the range of need-related experiences captured by the revised scales. Future research should explicitly distinguish between need satisfaction, low need satisfaction, and need frustration when adapting TENS-related instruments to AI-supported contexts.

Another limitation concerns the non-inclusion of relatedness dimensions in the Interface, Task, and Behavior spheres. Although relatedness was retained and validated in the Life sphere, the present study did not examine whether AI technologies may support or undermine relatedness at more specific levels of interaction and use. This decision was informed by pilot testing, in which participants reported difficulty interpreting the existing TENS relatedness items in relation to their use of generative AI tools, particularly when the items referred to interpersonal closeness, belonging, or meaningful connections with others. Nevertheless, conversational AI may produce relational or socially meaningful experiences, especially when users anthropomorphize the technology or develop a sense of connection with conversational agents (Airenti, 2015; Christoforakos et al., 2021). In addition, if AI-supported learning tools replace rather than complement human interaction, they may reduce opportunities for peer communication, teacher–student interaction, and social belonging, thereby potentially undermining students’ need for relatedness (Crawford et al., 2024). Future research should therefore examine relatedness more explicitly in AI-supported contexts, distinguishing between technology-mediated relatedness to other people and perceived relatedness with the technology itself. Recent technology-specific measures, such as the Basic Psychological Needs Scale for Technology Use (BPN-TU) (Moradbakhti et al., 2024), may provide useful reference points for further examining this distinction in AI-related settings.

Finally, although the revised scales demonstrated acceptable psychometric properties overall, several dimensions contained items with relatively modest factor loadings, particularly within the revised TENS-Task autonomy construct. These findings suggest that the relevant dimensions should be interpreted with caution until further validation is conducted. Future research could employ larger and more diverse samples, longitudinal designs, test–retest procedures, or behavioral indicators of AI use to provide additional evidence for the robustness and temporal stability of the TENS scales in AI-supported learning environments.

## 7 Conclusion

This study validated the five TENS scales derived from the METUX model within a Chinese university student sample. Through a sequential, multi-phase approach, including reliability analysis, CFA, and item-level refinement, this study examined the psychometric properties and structural validity of the original and revised versions of the TENS scales. Overall, the findings provide empirical support for the applicability of the five scales derived from the METUX model in non-English speaking educational settings. In particular, the revised TENS-Interface and TENS-Task scales demonstrated substantially improved model fit, internal consistency, and convergent validity, reinforcing the importance of iterative refinement and contextual sensitivity in scale adaptation across cultural settings. These validated tools provide a reliable foundation for future research on students’ psychological need satisfaction in AI-supported or technology-enhanced learning environments. Future research could extend this work by testing the TENS scales in diverse cultural and disciplinary contexts to further examine their generalizability and predictive utility.

## Data Availability

The original contributions presented in the study are included in the article/Supplementary material, further inquiries can be directed to the corresponding authors.

## Footnotes

1 Phase I: two institutes from Shaanxi province, and one from Zhejiang province.

2 One institute from Liaoning Province, and the other institute from Fujian Province.
