# Supplementary material for: Applying and validating the METUX model in Chinese higher education: a psychometric assessment of AI-based need satisfaction scales
Source: Front Psychol. 2026 Jul 3;17:1822374. doi: 10.3389/fpsyg.2026.1822374 (PMC13377021; doi:10.3389/fpsyg.2026.1822374)
Supplement: Supplementary file 1 [file Supplementary_file_1.docx]

Appendices

Appendix 1 ACTA Scale—English-Chinese Version

| **English version** | **中文版本** |
| --- | --- |
| Other people want me to use it. (external) | （ACTA1）我开始使用一些AI工具，是因为别人希望我去使用。（例如，学校、老师、父母、同学的推荐等） |
| I expected it will be interesting to use. (intrinsic) | （ACTA2）我开始使用一些AI工具，是因为我想着它用起来会有趣。 |
| I believe it could improve my life. (identified) | （ACTA3）我开始使用一些AI工具，是因为我相信它可以改善我的学习和生活。 |
| It will help me do something important to me. (identified) | （ACTA4）我开始使用一些AI工具，是因为它能帮助我完成一些对我而言重要的事情。 |
| I want others to know I use it. (introjected) | （ACTA5）我开始使用一些AI工具，是因为我希望别人知道我在使用AI。 |
| I will feel bad about myself (ashamed) if I didn't try it, because I feel I should be using the latest AI technology (introjected) | （ACTA6）如果我不去尝试使用AI，我会对自己感到失望，因为我觉得我应该尝试最新的AI技术。 |
| I think it would be enjoyable. (intrinsic) | （ACTA7）我开始使用一些AI工具，是因为我想着它会是一个令人愉快的尝试。 |
| I am required to use it (e.g., by my school, teacher, peers). (external) | （ACTA8）我开始使用一些AI工具，是因为我被要求使用它（例如因为学校或者老师的要求）。 |
| It is going to be of value to me in my life. (identified) | （ACTA9）我开始使用一些AI工具，是因为我认为它对我的生活是有价值的。 |
| It is going to be fun to use. (intrinsic) | （ACTA10）我开始使用一些AI工具，是因为我认为使用它会充满乐趣。 |
| I feel pressured to use it. (external) | （ACTA11）我开始使用一些AI工具，完全是迫于外界的压力而使用它。（例如，学校或者老师的硬性要求） |
| It will look good to others if I use it. (introjected) | （ACTA12）我开始使用一些AI工具，是因为我认为使用它会让我在别人眼中显得很好。 |
| I feel confident that I’ll be able to use AI effectively. | （ACTA13）我有信心能高效地使用AI, 所以我开始尝试使用它。 |
| The AI technology will be easy for me to use. | （ACTA14）我认为AI对我而言使用起来会很容易，所以我开始尝试使用它。 |

Appendix 2 TENS-Interface scale—English-Chinese Version

| English version | **中文版本** |
| --- | --- |
| **Competence** | **胜任力** |
| I feel very capable and effective at using the AI technology. | （IF1）在使用AI时，我觉得自己很熟练、很得心应手。 |
| I feel confident in my ability to use the AI technology. | （IF2）在使用AI时，我对自己的能力很有信心。 |
| Learning how to use the AI technology was difficult. (-) | （IF3）我发现有时候学会使用一些AI工具挺困难的。 |
| I found the interface and controls confusing. (-) | （IF4）我觉得有些AI工具的使用界面和操作让人搞不清楚。 |
| It wasn’t easy to use AI technology. (-) | （IF5）我发现有些AI工具使用起来不太简便。 |
| **Autonomy** | **自主性** |
| The AI technology provides me with useful options and choices | （IF6）在使用AI时，它可以为我提供很多有用的选择和功能。 |
| I can get the AI technology to do the things I want it to. | （IF7）在使用AI时，我能让它实现我想要的操作。 |
| I feel pressured by the AI technology. (-) | （IF8）我觉得使用AI时，它让我感到有压力。 |
| The AI technology feels intrusive (-) | （IF9）我觉得使用AI时，它干扰了我的自主操作。 |
| The AI technology feels controlling. (-) | （IF10）我觉得在使用AI时像是在被它主导，也就是让我觉得AI在控制我，而不是我在使用它。 |

Appendix 3 TENS-Task scale—English-Chinese Version

| **English version** | **中文版本** |
| --- | --- |
| **Competence** | **胜任力** |
| I feel confident in my ability to use AI to generate study ideas or solve problems. | （T1）我对自己使用AI获取学习思路或解决问题的能力感到自信。(例如，提问开放性问题，梳理重点、解释复杂概念等) |
| It’s easy to use AI to generate study ideas or solve problems. | （T2）使用AI获取学习思路或解决问题对我来说很容易。(例如，提问开放性问题，梳理重点、解释复杂概念等) |
| I find using AI to generate study ideas or solve problems too challenging. (-) | （T3）我觉得使用AI来获取学习思路或解决问题太有挑战性。(例如，提问开放性问题，梳理重点、解释复杂概念等) |
| I find using AI to generate study ideas or solve problems too difficult to do regularly (-) | （T4）我觉得使用AI获取学习思路或解决问题很难经常进行。(例如，提问开放性问题，梳理重点、解释复杂概念等) |
| **Autonomy** | **自主性** |
| I feel pressured to use AI to get study ideas or solve problems. (-) | （T5）我觉得自己在使用AI获取学习思路或解决问题时感到有压力。 |
| I only use AI to get study ideas or solve problems because I have to.(-) | （T6）我只是因为必须要用，才使用AI来获取学习思路或解决问题。 |
| I use AI tools to get study ideas or solve problems because other people want me to. (-) | （T7）我使用AI获取学习思路或解决问题，是因为别人希望我这么做。（例如，老师或者同学） |
| I will feel guilty if I don’t use AI to get study ideas or solve problems. (-) | （T8）如果我不使用AI来获取学习思路或解决问题，我会感到惭愧。 |

Appendix 4 TENS-Behaviour scale—English-Chinese Version

| **English version** | **中文版本** |
| --- | --- |
| **Autonomy** | **胜任力** |
| AI provides me with different options for engaging in my learning activities. | （B1）AI为我在参与学习活动时提供了多种选择方式。 |
| AI gives me freedom to engage in my learning activities the way I want | （B2）AI让我可以用我喜欢的方式去参与学习活动。 |
| AI helps me have more choice over how I engage in my learning activities. | （B3）AI让我对如何参与学习活动有了更多的选择权。 |
| **Competence** | **自主性** |
| AI helps me feel confident in my ability to engage in my learning activities. | （B4）AI让我对自己参与学习活动的能力更有信心。 |
| AI helps me engage in my learning activities effectively | （B5）AI帮助我更高效地参与学习活动。 |
| AI is useful for engaging in my learning activities | （B6）AI对我参与学习活动很有帮助。 |

Appendix 5 TENS-Life scale—English-Chinese Version

| **English version** | **中文版本** |
| --- | --- |
| **Autonomy** | **自主性** |
| Now that I use the AI technology, I feel pressured to use it more often than I’d like. (-) | （L1）自从我开始使用AI后，我常常会不自觉地比原本想的更频繁地使用它。 |
| I spend more time on the AI technology than I feel I should. (-) | （L2）我花在AI上的时间比我想象中的时间要多。 |
| The AI technology ends up making me do things I don’t want to do. (-) | （L3）AI让我花时间做了一些原本不在我计划内、也不是我真正想做的事情。 |
| The AI technology intrudes in my life. (-) | （L4）AI有点过度介入了我的生活。 |
| **Competence** | **胜任力** |
| Using AI technology has made me feel insecure about my abilities. (-) | （L5）自从使用AI后，让我对自己的能力产生了怀疑。 |
| Using AI technology has made me feel less capable in my life. (-) | （L6）自从使用AI后，让我觉得自己在生活中变得没那么能干了。 |
| Using AI technology has lowered my confidence. (-) | （L7）自从使用AI后，让我变得不那么自信了。 |
| **Relatedness** | **关联性** |
| Using AI technology has helped me feel a greater sense of belonging to a larger community | （L8）自从使用AI后，让我更有一种归属感，觉得自己是更大群体中的一员。 |
| Using AI technology has helped me feel close and connected with other people who are important to me. | （L9）自从使用AI后，让我感到自己与那些对我重要的人更加亲近和有连接感。 |
| Because of AI technology, I feel closer to some others. | （L10）因为AI这项技术，我觉得自己和一些人更亲近了。 |

Appendix 6 ACTA scale inter-item correlations

| Item Label | ACTA1 | ACTA2 | ACTA3 | ACTA4 | ACTA5 | ACTA6 | ACTA7 | ACTA8 | ACTA9 | ACTA10 | ACTA11 | ACTA12 | ACTA13 | ACTA14 |
| --- | --- | --- | --- | --- | --- | --- | --- | --- | --- | --- | --- | --- | --- | --- |
| ACTA1 | 1 |  |  |  |  |  |  |  |  |  |  |  |  |  |
| ACTA2 | .322** | 1 |  |  |  |  |  |  |  |  |  |  |  |  |
| ACTA3 | .283** | .391** | 1 |  |  |  |  |  |  |  |  |  |  |  |
| ACTA4 | .340** | .391** | .671** | 1 |  |  |  |  |  |  |  |  |  |  |
| ACTA5 | .342** | .181** | -0.023 | 0.1 | 1 |  |  |  |  |  |  |  |  |  |
| ACTA6 | .365** | .218** | 0.082 | .162** | .555** | 1 |  |  |  |  |  |  |  |  |
| ACTA7 | .285** | .513** | .627** | .590** | .170** | .162** | 1 |  |  |  |  |  |  |  |
| ACTA8 | .315** | .196** | 0.02 | .162** | .649** | .535** | .169** | 1 |  |  |  |  |  |  |
| ACTA9 | .279** | .540** | .571** | .581** | 0.065 | 0.091 | .697** | 0.075 | 1 |  |  |  |  |  |
| ACTA10 | .350** | .526** | .429** | .458** | .394** | .284** | .648** | .313** | .573** | 1 |  |  |  |  |
| ACTA11 | .290** | .162** | 0.005 | .167** | .676** | .503** | .160** | .778** | 0.087 | .308** | 1 |  |  |  |
| ACTA12 | .294** | .236** | 0.097 | .180** | .600** | .512** | .200** | .659** | .180** | .320** | .579** | 1 |  |  |
| ACTA13 | .303** | .626** | .396** | .417** | .192** | .171** | .521** | .208** | .493** | .382** | .167** | .242** | 1 |  |
| ACTA14 | .327** | .539** | .431** | .424** | .175** | .205** | .635** | .195** | .628** | .511** | .162** | .290** | .534** | 1 |
| Note. Intrinsic: ACTA2, ACTA7, ACTA10; Identified: ACTA3, ACTA4, ACTA9; Introjected: ACTA5, ACTA6, ACTA12; External: ACTA1, ACTA8, ACTA11; Competence: ACTA13, ACTA14 | | | | | | | | | | | | | | |

Appendix 7 TENS-Interface scale inter-item correlations

| Item Label | IF1 | IF2 | IF3 | IF4 | IF5 | IF6 | IF7 | IF8 | IF9 | IF10 |
| --- | --- | --- | --- | --- | --- | --- | --- | --- | --- | --- |
| IF1 | 1 |  |  |  |  |  |  |  |  |  |
| IF2 | .562** | 1 |  |  |  |  |  |  |  |  |
| IF3 | -0.061 | -.175** | 1 |  |  |  |  |  |  |  |
| IF4 | 0.01 | -.128* | .600** | 1 |  |  |  |  |  |  |
| IF5 | -.202** | -.284** | .548** | .478** | 1 |  |  |  |  |  |
| IF6 | .441** | .577** | -.235** | -.242** | -.438** | 1 |  |  |  |  |
| IF7 | .481** | .635** | -.219** | -.172** | -.361** | .663** | 1 |  |  |  |
| IF8 | -0.027 | -.112* | .463** | .547** | .353** | 0.045 | -0.109 | 1 |  |  |
| IF9 | -0.045 | -.149** | .525** | .549** | .345** | -0.094 | -.206** | .663** | 1 |  |
| IF10 | -0.037 | -0.092 | .462** | .523** | .241** | 0.012 | -.111* | .639** | .711** | 1 |
| Note. Competence: IF1-IF5; Autonomy: IF6-IF10. | | | | | | | | | | |

Appendix 8 TENS-Task scale inter-item correlations

| Item Label | T1 | T2 | T3 | T4 | T5 | T6 | T7 | T8 |
| --- | --- | --- | --- | --- | --- | --- | --- | --- |
| T1 | 1 |  |  |  |  |  |  |  |
| T2 | .519** | 1 |  |  |  |  |  |  |
| T3 | -.284** | -.259** | 1 |  |  |  |  |  |
| T4 | -.249** | -0.092 | .390** | 1 |  |  |  |  |
| T5 | -.183** | -0.059 | .371** | .593** | 1 |  |  |  |
| T6 | -.255** | -0.096 | .310** | .579** | .676** | 1 |  |  |
| T7 | -.135* | -0.06 | .390** | .550** | .746** | .660** | 1 |  |
| T8 | -.260** | -.151** | .307** | .421** | .622** | .545** | .600** | 1 |
| Note. Competence: T1-T4; Autonomy: T5-T8. | | | | | | | | |

Appendix 9 TENS-Behaviour scale inter-item correlations

| Item Label | B1 | B2 | B3 | B4 | B5 | B6 |
| --- | --- | --- | --- | --- | --- | --- |
| B1 | 1 |  |  |  |  |  |
| B2 | .498** | 1 |  |  |  |  |
| B3 | .712** | .586** | 1 |  |  |  |
| B4 | .398** | .516** | .474** | 1 |  |  |
| B5 | .579** | .617** | .654** | .632** | 1 |  |
| B6 | .545** | .709** | .631** | .622** | .734** | 1 |
| Note. Competence: B1-B3; Autonomy: B4-B6. | | | | | | |

Appendix 10 TENS-Life scale inter-item correlations

| Item Label | L1 | L2 | L3 | L4 | L5 | L6 | L7 | L8 | L9 | L10 |
| --- | --- | --- | --- | --- | --- | --- | --- | --- | --- | --- |
| L1 | 1 |  |  |  |  |  |  |  |  |  |
| L2 | .437** | 1 |  |  |  |  |  |  |  |  |
| L3 | .274** | .537** | 1 |  |  |  |  |  |  |  |
| L4 | .267** | .573** | .624** | 1 |  |  |  |  |  |  |
| L5 | .216** | .516** | .595** | .656** | 1 |  |  |  |  |  |
| L6 | .297** | .467** | .510** | .632** | .627** | 1 |  |  |  |  |
| L7 | .204** | .507** | .525** | .689** | .588** | .679** | 1 |  |  |  |
| L8 | -.287** | -.551** | -.451** | -.510** | -.445** | -.422** | -.506** | 1 |  |  |
| L9 | -.277** | -.470** | -.335** | -.468** | -.479** | -.391** | -.412** | .577** | 1 |  |
| L10 | -.256** | -.404** | -.354** | -.414** | -.315** | -.325** | -.433** | .511** | .556** | 1 |
| Note. Autonomy: L1-L4; Competence: L5-L7; Relatedness: L8-L10. | | | | | | | | | | |

Appendix 11 Model Fit Indices for CFA on Each Dimension of the TENS Scales

| **Model** | | **χ2** | **df** | **χ2/df** | **CFI** | **TLI** | **RMSEA** | **(90%CI)** | **SRMR** | **GFI** |
| --- | --- | --- | --- | --- | --- | --- | --- | --- | --- | --- |
| ACTA | Intrinsic | 0.00 | 0 | 0.00 | 1.00 | 1.00 | 0.56 | (.505, .611) | 0.00 | 1.00 |
|  | Identified | 0.00 | 0 | 0.00 | 1.00 | 1.00 | 0.60 | (.552, .658) | 0.00 | 1.00 |
|  | Introjected | 0.00 | 0 | 0.00 | 1.00 | 1.00 | 0.54 | (.490, .596) | 0.00 | 1.00 |
|  | External | 0.00 | 0 | 0.00 | 1.00 | 1.00 | 0.59 | (.534, .640) | 0.00 | 1.00 |
|  | Competence | — | — | — | — | — | — | — | — | — |
| TENS- Interface | Competence | 135.28 | 5 | 27.06 | 0.69 | 0.38 | 0.29 | (.245, .328) | 0.15 | 0.86 |
|  | Autonomy | 447.21 | 5 | 89.44 | 0.31 | -0.38 | 0.53 | (.486, .568) | 0.29 | 0.64 |
| TENS-Task | Competence | 40.22 | 2 | 20.11 | 0.80 | 0.40 | 0.25 | (.182, .313) | 0.09 | 0.94 |
|  | Autonomy | 0.04 | 2 | 0.02 | 1.00 | 1.01 | 0.00 | (.000, .000) | 0.00 | 1.00 |
| TENS-Behaviour | Competence | 0.00 | 0 | 0.00 | 1.00 | 1.00 | 0.68 | (.623, .729) | 0.00 | 1.00 |
|  | Autonomy | 0.00 | 0 | 0.00 | 1.00 | 1.00 | 0.62 | (.564, .670) | 0.00 | 1.00 |
| TENS-Life | Competence | 0.00 | 0 | 0.00 | 1.00 | 1.00 | 0.63 | (.578, .685) | 0.00 | 1.00 |
|  | Autonomy | 21.66 | 2 | 10.83 | 0.95 | 0.84 | 0.18 | (.114, .246) | 0.05 | 0.97 |
|  | Relatedness | 0.00 | 0 | 0.00 | 1.00 | 1.00 | 0.53 | (.479, .586) | 0.00 | 1.00 |

Appendix 12 Revised TENS-Interface scale (English-Chinese Version)

| **English version** | **中文版本** |
| --- | --- |
| **Competence** | **胜任力** |
| I feel very capable and effective at using AI technology. | （IF1）我觉得自己能够熟练、得心应手的使用AI。 |
| I feel confident in my ability to use AI technology. | （IF2）我对自己使用AI的能力有信心。 |
| I learned how to use AI technology easily. | （IF3）我很容易就学会了如何使用AI工具。 |
| I found the interface and controls clear and easy to understand. | （IF4）我觉得AI工具的界面和操作清晰且容易理解。 |
| It was easy to use AI technology. | （IF5）我发现AI工具使用起来是容易的。 |
| **Autonomy** | **自主性** |
| The AI technology provides me with useful options and choices | （IF6）在使用AI时，它可以为我提供很多有用的选择和功能。 |
| I can get the AI technology to do the things I want it to. | （IF7）在使用AI时，我能让它实现我想要的操作。 |
| The AI technology allows me to engage at my own pace. | （IF8）在使用AI时，它允许我以自己的节奏使用或者参与。 |
| The AI technology respects my personal space | （IF9）在使用AI时，它尊重我的个人空间。 |
| The AI technology allows me to stay in control. | （IF10）在使用AI时，我始终掌握主动权。 |

Appendix 13 Revised TENS-Task scale (English-Chinese Version)

| **English version** | **中文版本** |
| --- | --- |
| **Competence** | **胜任力** |
| I feel confident in my ability to use AI to generate study ideas or solve problems. | （T1）我对自己使用AI获取学习思路或解决问题的能力感到自信。(例如，提问开放性问题，梳理重点、解释复杂概念等) |
| It’s easy to use AI to generate study ideas or solve problems. | （T2）使用AI获取学习思路或解决问题对我来说是容易的。(例如，提问开放性问题，梳理重点、解释复杂概念等) |
| I find using AI to generate study ideas or solve problems manageable and within my ability. | （T3）我觉得我有能力使用AI来产生学习想法或解决问题。(例如，提问开放性问题，梳理重点、解释复杂概念等) |
| I find using AI to generate study ideas or solve problems easy to do regularly. | （T4）我觉得经常使用AI来产生学习想法或解决问题是件轻松的事。(例如，提问开放性问题，梳理重点、解释复杂概念等) |
| **Autonomy** | **自主性** |
| I feel pressured to use AI to get study ideas or solve problems. (-) | （T5）我迫于压力去使用AI获取学习思路或解决问题。 |
| I only use AI to get study ideas or solve problems because I have to.(-) | （T6）只有当我必须要使用AI时，我才使用它来获取学习思路或解决问题。 |
| I use AI tools to get study ideas or solve problems because other people want me to. (-) | （T7）我使用AI获取学习思路或解决问题，是因为别人希望我这么做。（例如，老师或者同学） |
| I will feel guilty if I don’t use AI to get study ideas or solve problems. (-) | （T8）如果我不使用AI来获取学习思路或解决问题，我会感到不安。 |

Appendix 14 Revised TENS-Interface scale inter-item correlations

| Item Lable | IF1 | IF2 | IF3 | IF4 | IF5 | IF6 | IF7 | IF8 | IF9 | IF10 |
| --- | --- | --- | --- | --- | --- | --- | --- | --- | --- | --- |
| IF1 | 1 |  |  |  |  |  |  |  |  |  |
| IF2 | .636** | 1 |  |  |  |  |  |  |  |  |
| IF3 | .547** | .466** | 1 |  |  |  |  |  |  |  |
| IF4 | .562** | .472** | .716** | 1 |  |  |  |  |  |  |
| IF5 | .594** | .591** | .638** | .643** | 1 |  |  |  |  |  |
| IF6 | .502** | .412** | .597** | .560** | .605** | 1 |  |  |  |  |
| IF7 | .577** | .504** | .548** | .470** | .528** | .582** | 1 |  |  |  |
| IF8 | .529** | .457** | .575** | .637** | .537** | .640** | .639** | 1 |  |  |
| IF9 | .546** | .523** | .516** | .484** | .499** | .517** | .621** | .556** | 1 |  |
| IF10 | .567** | .508** | .504** | .483** | .514** | .513** | .679** | .578** | .633** | 1 |

Appendix 15 Revised TENS-Task scale inter-item correlations

| Item Label | T1 | T2 | T3 | T4 | T5 | T6 | T7 | T8 |
| --- | --- | --- | --- | --- | --- | --- | --- | --- |
| T1 | 1 |  |  |  |  |  |  |  |
| T2 | .674** | 1 |  |  |  |  |  |  |
| T3 | .595** | .694** | 1 |  |  |  |  |  |
| T4 | .568** | .567** | .565** | 1 |  |  |  |  |
| T5 | .310** | .271** | 0.128 | 0.139 | 1 |  |  |  |
| T6 | .414** | .464** | .335** | .287** | .286** | 1 |  |  |
| T7 | .191** | .208** | 0.08 | .145* | .522** | .160* | 1 |  |
| T8 | .159* | .214** | 0.077 | 0.125 | .430** | 0.087 | .305** | 1 |

Appendix 16 Model Fit Indices for CFA on Each Dimension of the Modified TENS Scales

| **Model** | | **χ2** | **df** | **χ2/df** | **CFI** | **TLI** | **RMSEA** | **SRMR** | **GFI** |
| --- | --- | --- | --- | --- | --- | --- | --- | --- | --- |
| TENS- Interface | Competence | 34.74 | 5 | 6.95 | 0.94 | 0.87 | 0.18 | 0.05 | 0.93 |
|  | Autonomy | 14.20 | 5 | 2.84 | 0.98 | 0.96 | 0.10 | 0.03 | 0.97 |
| TENS-Task | Competence | 3.28 | 2 | 1.64 | 1.00 | 0.99 | 0.06 | 0.02 | 0.99 |
|  | Autonomy | 1.39 | 2 | 0.70 | 1.00 | 1.02 | 0.00 | 0.02 | 1.00 |

Appendix 17 CFA Fit Indices for the Five TENS Scales

| **Model** | **χ2** | **df** | **χ2/df** | **CFI** | **TLI** | **RMSEA** | **SRMR** | **GFI** | **ECVI** |
| --- | --- | --- | --- | --- | --- | --- | --- | --- | --- |
| ACTA | 230.31 | 65.00 | 3.54 | 0.93 | 0.90 | 0.09 | 0.09 | 0.91 | — |
| TENS-Interface(Original) | 645.44 | 34.00 | 18.98 | 0.60 | 0.46 | 0.24 | 0.20 | 0.67 | 2.16 |
| TENS-Interface(Revised) | 77.19 | 33.00 | 2.34 | 0.96 | 0.95 | 0.08 | 0.04 | 0.92 | 0.65 |
| TENS-Task(Original) | 138.93 | 19.00 | 7.31 | 0.89 | 0.83 | 0.14 | 0.09 | 0.90 | 0.54 |
| TENS-Task(Revised) | 57.19 | 19.00 | 3.01 | 0.93 | 0.89 | 0.10 | 0.10 | 0.94 | 0.49 |
| TENS-Behaviour | 67.11 | 8.00 | 8.39 | 0.95 | 0.90 | 0.15 | 0.05 | 0.93 | — |
| TENS-Life | 120.58 | 32.00 | 3.77 | 0.94 | 0.92 | 0.09 | 0.05 | 0.93 | — |
